# Assembly and Turnover of Caveolae: What Do We Really Know?

**DOI:** 10.3389/fcell.2016.00068

**Published:** 2016-06-27

**Authors:** Bing Han, Courtney A. Copeland, Ajit Tiwari, Anne K. Kenworthy

**Affiliations:** ^1^Department of Molecular Physiology and Biophysics, Vanderbilt University School of MedicineNashville, TN, USA; ^2^Department of Cell and Developmental Biology, Vanderbilt University School of MedicineNashville, TN, USA; ^3^Epithelial Biology Program, Vanderbilt University School of MedicineNashville, TN, USA; ^4^Chemical and Physical Biology Program, Vanderbilt UniversityNashville, TN, USA

**Keywords:** caveolae, caveolin-1, GFP, trafficking, degradation, breast cancer, pulmonary arterial hypertension, congenital generalized lipodystrophy

## Abstract

In addition to containing highly dynamic nanoscale domains, the plasma membranes of many cell types are decorated with caveolae, flask-shaped domains enriched in the structural protein caveolin-1 (Cav1). The importance of caveolae in numerous cellular functions and processes has become well-recognized, and recent years have seen dramatic advances in our understanding of how caveolae assemble and the mechanisms control the turnover of Cav1. At the same time, work from our lab and others have revealed that commonly utilized strategies such as overexpression and tagging of Cav1 have unexpectedly complex consequences on the trafficking and fate of Cav1. Here, we discuss the implications of these findings for current models of caveolae biogenesis and Cav1 turnover. In addition, we discuss how disease-associated mutants of Cav1 impact caveolae assembly and outline open questions in this still-emerging area.

## Introduction

In addition to containing nanoclusters of proteins and lipids, the surface of many cell types also contain relatively stable flask-shaped invaginations that are 50–100 nm in diameter known as caveolae. Initially discovered nearly 60 years ago in the plasma membranes of endothelial cells of blood capillaries by electron microscopy, caveolae have been a target of scientific investigation for decades (Palade, 1953). The discovery of the first caveolae-associated protein caveolin-1 (Cav1) almost 40 years after the discovery of caveolae has greatly facilitated research into the structural and functional aspects of caveolae (Kurzchalia et al., 1992; Rothberg et al., 1992). To date, caveolae have been identified in a variety of tissues and cell types including endothelial cells, smooth muscle cells, fibroblasts, myoblasts, and adipocytes, among others (Hansen et al., 2013; Parton and del Pozo, 2013), and the importance of a series of accessory proteins in sculpting caveolae and regulating their dynamics is also now recognized (Hill et al., 2008; Hansen and Nichols, 2010; Hansen et al., 2011; Moren et al., 2012; Stoeber et al., 2012; Ariotti and Parton, 2013; Ludwig et al., 2013; Kovtun et al., 2014, 2015). It is also now clear that once formed, caveolae can flatten in response to membrane stretch and thus serve as membrane reservoirs (Gervasio et al., 2011; Sinha et al., 2011).

Unlike the more controversial case of lipid rafts (Owen et al., 2012; Kraft, 2013; LaRocca et al., 2013; Sevcsik and Schutz, 2016), caveolae are relatively stable structures and also thus readily detectable by conventional fluorescence and electron microscopy approaches. In addition, their presence in cells absolutely depends on the expression of Cav1, making them amenable to a range of biochemical and biophysical analyses as well as studies in animal models (Drab et al., 2001; Razani et al., 2001; Le Lay and Kurzchalia, 2005). Through these varied approaches, the importance of caveolae in numerous cellular functions and processes has become well-recognized, and are thought to include roles in signal transduction, endocytosis, pathogen invasion, lipid homeostasis, and mechanotransduction (Parton and Simons, 2007; Hansen and Nichols, 2010; Ariotti and Parton, 2013; Parton and del Pozo, 2013; Cheng and Nichols, 2016). Furthermore, Cav1 and other caveolins have been implicated several pulmonary and vascular diseases, myopathies, lipodystrophies, and cancers (Hayashi et al., 2001; Razani and Lisanti, 2001; Cao et al., 2008; Kim et al., 2008; Mercier et al., 2009; Austin et al., 2012; Ariotti and Parton, 2013; Garg et al., 2015; Martinez-Outschoorn et al., 2015).

Given the importance of caveolae in both health and disease, it is critical to gain a clear understanding of how caveolae form and the mechanisms responsible for the turnover of their components. In this mini-review, we summarize current knowledge in these areas, including the unexpectedly complex consequences that overexpression and tagging of Cav1 can have on the trafficking and fate of Cav1 and caveolae biogenesis. In addition, we discuss how disease-associated mutants of Cav1 impact caveolae assembly and turnover and outline open questions in this emerging area.

## What conditions are necessary for caveolae to form correctly?

It is widely accepted that the assembly of caveolae requires the expression of Cav1 (Drab et al., 2001; Razani et al., 2001). A 178 amino acid-long protein, Cav1 is anchored to the membrane by an intra-membrane region that assumes a hairpin-like topology. The Cav1 protein contains four domains: the N-terminal domain (residues 1-81), scaffolding domain (CSD, residues 82-101), transmembrane domain (TMD, residues 102-134), and C-terminal domain (residues 135-178) (Root et al., 2015). The transmembrane domain is composed of two α-helices separated by three residue linker region containing a proline (P110) that induces a ~50° angle between the two α-helices (Root et al., 2015). This allows Cav1 to adopt a hairpin topology in the lipid bilayer such that both N- and C- termini are exposed to the cytoplasmic interior of the cell (Root et al., 2015). To date, however, the three dimensional structure of Cav1 remains unknown.

**Figure 1:**
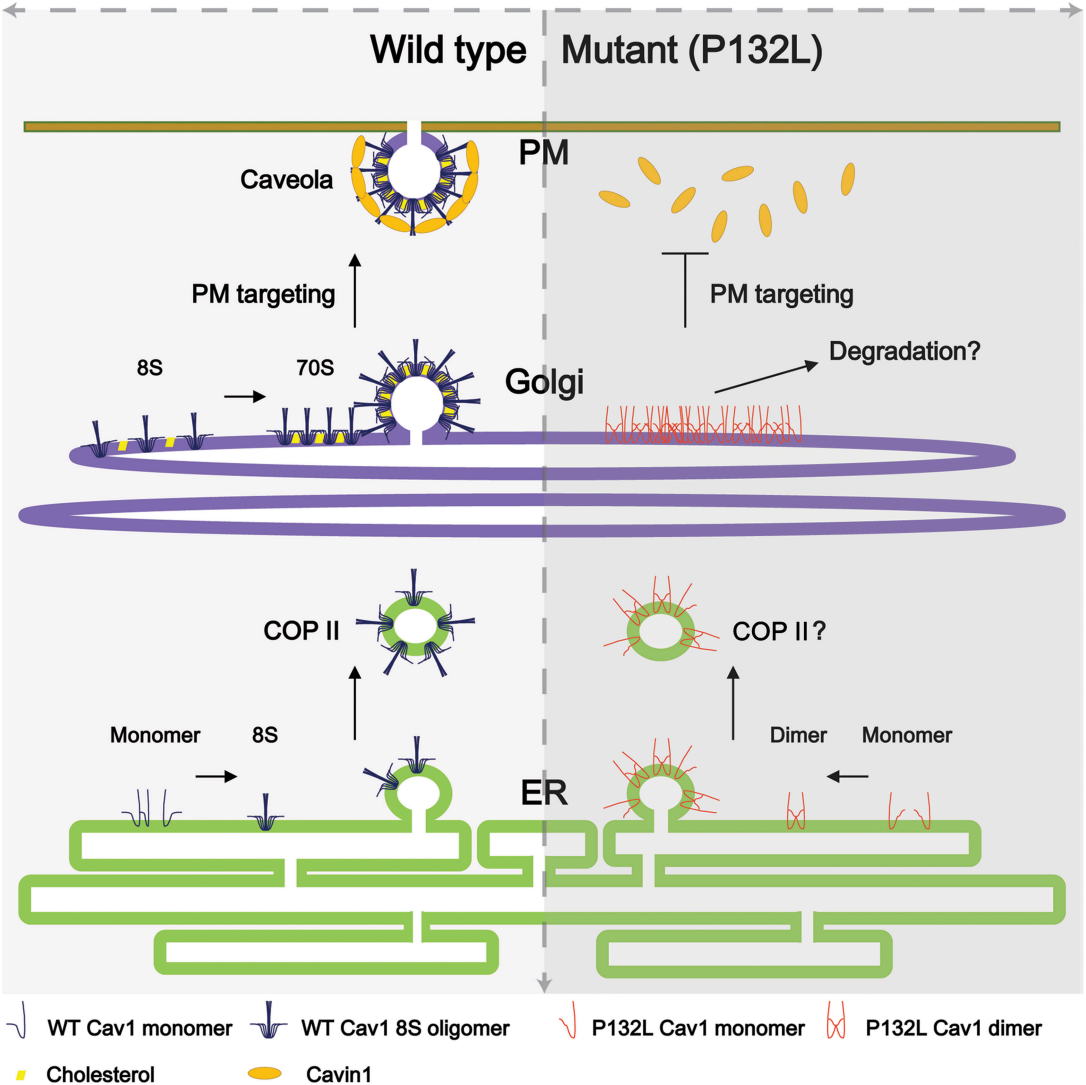
**Current model of caveolae biogenesis**. (**Left**) Newly synthesized wild type Cav1 undergoes a series of oligomerization events as it passes through the secretory pathway. At the plasma membrane, accessory proteins interact with Cav1 complexes to form mature caveolae. (**Right**) In contrast, a breast-cancer associated mutant of Cav1, Cav1-P132L, is unable to oligomerize correctly and accumulates in the Golgi complex, where it is likely targeted for degradation. For simplicity, not all caveolae accessory proteins are illustrated here.

Cav1 is synthesized in the endoplasmic reticulum and undergoes a complicated series of oligomerization and trafficking events well before reaching the plasma membrane (Figure 1). Newly synthesized Cav1 is quickly organized into Cav1/Cav2 (caveolin-2) hetero-oligomers that contains 14-16 monomers (Monier et al., 1995; Sargiacomo et al., 1995) and partition as an 8S complex on sucrose gradients (Hayer et al., 2010a). This 8S-oligomerization step appears to be pivotal for the proper assembly of caveolae, because forms of Cav1 that fail to oligomerize are unable to independently assemble into caveolae (Mora et al., 1999; Lee et al., 2002; Ren et al., 2004; Shatz et al., 2010). Thereafter, 8S complexes are transported to the Golgi complex in a COPII-dependent mechanism where they serve as the subunits necessary for the assembly of filament-like 70S complexes that become enriched in cholesterol and lose their diffusional mobility. The cholesterol-rich membranes containing 70S Cav1 complexes are then transported to the cell surface (Hayer et al., 2010a).

At the plasma membrane, several accessory proteins are subsequently recruited to caveolin complexes to facilitate caveolae formation and assist in sculpting caveolar membranes as well as regulate caveolae dynamics. They include members of the cavin gene family, pacsin-2, and EHD-2 (Aboulaich et al., 2004; Hill et al., 2008; Hansen and Nichols, 2010; Hansen et al., 2011; Moren et al., 2012; Stoeber et al., 2012; Ariotti and Parton, 2013; Ludwig et al., 2013; Kovtun et al., 2014, 2015). Cavin-1 plays an important role in forming caveolae, as cavin-1 knock-down significantly reduces caveolae number in both mammalian cells and zebrafish (Hill et al., 2008) and cavin-1 knockout mice lack caveolae altogether (Liu et al., 2008). Additional cavin family members have also been identified, and recent studies have elucidated the organization and structure of multiple cavin-containing complexes (Hayer et al., 2010a; Ludwig et al., 2013; Gambin et al., 2014; Kovtun et al., 2014, 2015). These findings have been reviewed in detail elsewhere (Kovtun et al., 2015) and will not be discussed further here. EHD-2 is thought to help confine caveolae and reduce mobility at the plasma membrane through interactions with actin (Moren et al., 2012; Stoeber et al., 2012). Pacsin-2, which contains a membrane curvature-associated F-BAR domain, has also been reported to be recruited to and assist in sculpting caveolae (Hansen et al., 2011; Senju et al., 2011). Furthermore, post-translational modifications of Cav1 such as palmitoylation and phosphorylation also regulate steps in caveolae assembly and caveolae structure (Monier et al., 1996; Nomura and Fujimoto, 1999; Zimnicka et al., 2016). However, expression of Cav1 in a bacterial expression system can drive the formation of heterologous caveolae. Thus, Cav1 itself is capable of inducing membrane curvature in some membrane environments, even without the help of accessory proteins (Walser et al., 2012; Ariotti et al., 2015).

The use of fluorescent protein-tagged forms of Cav1 has made it possible to assess caveolae biogenesis and dynamics. Such experiments have often been carried out by expressing low levels of Cav1 in Cav1^−∕−^ mouse embryonic fibroblasts (Kirkham et al., 2008; Ariotti et al., 2015) or more recently at endogenous expression levels in genome-edited cell lines (Shvets et al., 2015). However, a large literature also exists where Cav1 has been studied in the context of overexpression systems. One potential caveat of such studies is that both overexpression and tagging strategies can interfere with caveolae biogenesis (Parton and del Pozo, 2013). For example, it has been reported that in some cell types, after a few hours of expression overexpressed Cav1 fails to co-localize with endogenous Cav1, implying that exogenous Cav1 is not always incorporated into caveolae (Hayer et al., 2010b). Indeed, caveolin-enriched organelles termed “caveosomes” were later shown to arise as a consequence of the accumulation of overexpressed caveolin in late endosomal structures (Pelkmans et al., 2001; Hayer et al., 2010b).

Studies from our own group further have revealed that the behavior of overexpressed Cav1 also depends on the type of the tag (Hanson et al., 2013; Han et al., 2015). In COS-7 cells, for example, Cav1-GFP strongly accumulates in a perinuclear compartment (Hanson et al., 2013) in the form of irregular aggregates that contain little if any endogenous Cav1 (Han et al., 2015). The behavior of Cav1-mCherry differs dramatically from that of Cav1-GFP in the same cell line, both in terms of its subcellular localization (Hanson et al., 2013) and biochemical properties (Han et al., 2015). Furthermore, the degree to which Cav1-GFP accumulates intracellularly depends on the cell type in which it is expressed (Hanson et al., 2013). Thus, the ability of Cav1 to form oligomers and traffic correctly to the plasma membrane is heavily dependent on how the protein is tagged as well as the cellular environment, pointing to the exquisitely sensitive nature of caveolae assembly.

## What mechanisms are responsible for the turnover of Cav1 and caveolae?

Cav1 is known to be a relatively long-lived protein; estimates of the half-life of endogenous Cav1 from metabolic labeling studies range from 5 to 36 h (Conrad et al., 1995; Forbes et al., 2007; Hayer et al., 2010b). Turnover of Cav1 is accelerated under conditions that compromise caveolar assembly and/or destabilize 70S caveolar scaffolds (Hayer et al., 2010b). Under these conditions, Cav1 is ubiquitinated and targeted to endosomal sorting complex required for transport (ESCRT) machinery via intraluminal vesicles of multi-vesicular bodies and subsequently is degraded within lysosomes (Hayer et al., 2010b). Thus, under these conditions Cav1 behaves as endocytic cargo that is targeted to early endosomes and follows a classical endocytic pathway leading to degradation.

More recent evidence has revealed additional cellular machinery involved in Cav1 turnover by this pathway. One major contributor is Valosin Containing Protein (VCP/p97), an AAA-ATPase that functions in processing of ubiquitinated cellular proteins. Along with its cofactor UBXD1, VCP binds to monoubiquitinated Cav1 on endosomes and in turn influences trafficking, endosomal sorting, and degradation of Cav1 (Ritz et al., 2011). The ubiquitination events required for targeting Cav1 into this pathway occur at the N-terminal region of the protein (Kirchner et al., 2013). Turnover of ubiquitinated Cav1 is aided by the Ankrd13 proteins, which contain a ubiquitin interacting motif that bind to polyubiquinated Cav1 oligomers on endosomes (Burana et al., 2016). While these studies have defined a distinct pathway that controls the turnover of Cav1, there are hints in the literature that additional machinery and mechanisms involved in Cav1 turnover remain to be discovered (Austin et al., 2012; Bakhshi et al., 2013; Cha et al., 2015; Mougeolle et al., 2015; Schrauwen et al., 2015).

## How do disease-associated mutations affect caveolae assembly and turnover?

Cav1 has been implicated as a key player in a number of human diseases, and several disease-associated mutations in Cav1 have been identified (Hayashi et al., 2001; Razani and Lisanti, 2001; Cohen et al., 2004; Cao et al., 2008; Kim et al., 2008; Mercier et al., 2009; Austin et al., 2012; Ariotti and Parton, 2013; Garg et al., 2015; Martinez-Outschoorn et al., 2015). Perhaps the best known example is Cav1-P132L, originally identified as a somatic mutation associated with breast cancer (Hayashi et al., 2001). Although, the frequency with which this mutation occurs in humans has been highly debated (Hayashi et al., 2001; Lee et al., 2002; Koike et al., 2010; Lacroix-Triki et al., 2010; Ferraldeschi et al., 2012; Patani et al., 2012), Cav1-P132L has become a useful model for studying the behavior of mistrafficked forms of Cav1. This is because unlike wild type Cav1, Cav1-P132L typically localizes to the perinuclear region in a compartment proposed to correspond to the Golgi complex and does not form caveolae (Lee et al., 2002). Furthermore, Cav1-P132L primarily exists as monomer or dimer instead of the typical oligomers of wild type Cav1 observed in the cell (Lee et al., 2002; Ren et al., 2004; Hayer et al., 2010a; Rieth et al., 2012; Han et al., 2015). These features of Cav1-P132L differ substantially from the behavior of wild type Cav1 (Figure 1).

Interestingly, Cav1-P132L can also impact the behavior of wild type Cav1. In one of the earliest studies of Cav1-P132L, co-expression of Cav1-P132L with wild type Cav1 was shown to lead to a loss of wild type Cav1's affinity for detergent resistant membranes as well as to trap wild type Cav1 together with Cav1-P132L in a perinuclear compartment. Based on these findings, it was concluded that Cav1-P132L behaves in a dominant-negative manner, thereby interfering with caveolae formation (Lee et al., 2002). However, another study found that when co-expressed with wild type Cav1, Cav1-P132L had no effect on the localization of wild type Cav1 in FRT cells even though the mutant protein was localized in a perinuclear compartment (Ren et al., 2004). A different group showed that the number of caveolae increased upon stable expression of Cav1-P132L in H1299 cells, a cell line derived from human non-small cell carcinoma that endogenously expresses wild type Cav1 (Shatz et al., 2010). Thus, conflicting evidence exists as to how Cav1-P132L impacts caveolae assembly and function.

Why these behaviors of Cav1-P132L differ so much across studies is not yet clear. One potential clue comes from our recent observation that simply overexpressing Cav1-GFP causes a large fraction of the protein to be targeted to a perinuclear structure in COS-7 cells (Hanson et al., 2013). Furthermore, forms of Cav1 that were targeted to the plasma membrane when expressed separately became trapped intracellularly when they were co-expressed with Cav1-GFP (Hanson et al., 2013). Thus, in this system Cav1-GFP mimics the dominant negative trafficking defect originally reported for the Cav1-P123L mutant (Lee et al., 2002). Further, we observed that the majority of Cav1-GFP was degraded within 5 h, suggesting it may be improperly folded and thus targeted for degradation (Hanson et al., 2013). These findings raise the possibility that the dominant negative behavior reported for Cav1-P132L might at least in part be the result of misfolding induced by a combination of tagging and overexpression. They also raise questions about the identity of the perinuclear compartment that Cav1-GFP and Cav1-P132L accumulate in. In the case of Cav1-P132L, this compartment was originally proposed to correspond to the Golgi complex (Lee et al., 2002). However, given that perinuclear Cav1-GFP forms irregular aggregates, another possibility is that Cav1-GFP associates with aggresomes, structures that form in response to the accumulation of protein aggregates too large to be degraded by the proteasome (Wojcik et al., 1996; Johnston et al., 1998; Kopito, 2000; Garcia-Mata et al., 2002; Hyttinen et al., 2014). If this is the case, it would have important consequences for our current understanding of trafficking defects ascribed to mutant forms of both Cav1 and other caveolin family members, including a dominant negative P104L mutation in caveolin-3 associated with muscular dystrophy that corresponds to the P132L mutation in Cav1 (Carozzi et al., 2002; Sotgia et al., 2003; Pol et al., 2005; Cai et al., 2009).

In addition to Cav1-P132L, in recent years several additional disease-associated mutants of Cav1 have been identified, including one homozygous null mutation and three heterozygous frameshift mutations in the *Cav1* gene identified in patients with pulmonary arterial hypertension (PAH), lipodystrophy, or both (Kim et al., 2008; Austin et al., 2012; Garg et al., 2015; Schrauwen et al., 2015). The first mutation, c.G112T (p.E38X), is linked to lipodystrophy and leads to a complete loss of Cav1 protein expression (Kim et al., 2008). Two of the frameshift mutations, c.474delA (p.P158P fsX22), and c.473delC (p.P158H fsX22), generate a novel 21 amino acid-long C-terminus beyond amino acid position 158 associated with PAH (Austin et al., 2012). The third non-sense mutation, c.479_480delTT (p.F160X), introduces a premature stop codon that results in a truncated mutant protein lacking the last 19 amino acids of wild type Cav1 C-terminus. Interestingly, this mutation is associated with both PAH and congenital generalized lipodystrophy (Garg et al., 2015; Schrauwen et al., 2015).

How these mutant forms of Cav1 contribute to the development of PAH and/or congenital generalized lipodystrophy is not yet clear. However, one notable similarity shared by P158P/H and F160X is that they occur in the distal C-terminus of Cav1. This domain of Cav1 is thought to be important for Cav1 homo-oligomerization, Golgi-plasma membrane trafficking, and DRM association (Song et al., 1997; Machleidt et al., 2000). Initial studies in patient skin fibroblasts show that the presence of either P158P fsX22 or the truncation mutant F160X lead to decreased Cav1 protein levels (Austin et al., 2012; Schrauwen et al., 2015). It is thus possible that at least some of the newly identified PAH-associated Cav1 mutants are targeted for degradation, and may also function as dominant negatives against wild type Cav1. Caveolae assembly appears to be at least partially preserved for the case of the F160X mutation (Garg et al., 2015), although pathway analysis indicates its expression impacts signaling pathways that are important adipose tissue homeostasis (Schrauwen et al., 2015). It will be interesting to determine whether caveolae form correctly for the P158P mutants and whether Cav1 follows a conventional trafficking and degradative pathway in these patients.

## Conclusion

In summary, our understanding of how Cav1 assembles to form caveolae and is turned over outside of caveolae has increased tremendously over the past few years, yet is far from complete. A clear model of caveolae biogenesis has emerged, but additional work is needed to understand how disease-associated Cav1 mutants impact this process. Indeed, how wild type Cav1 itself is packed into caveolae is not yet entirely clear. How cells dispose of Cav1 in response to stress, and whether similar or different mechanisms are utilized to target various disease-associated mutants of Cav1 for degradation also remain to be more fully investigated. Some of these processes may be mimicked by overexpression of tagged forms of Cav1. Thus, further investigation of what may at first glance appear to be an artifact of tissue culture may ultimately reveal mechanisms that are of physiological and/or pathophysiological importance. Finally, it is important to recognize that a consensus model for how caveolae function does not yet exist (Cheng and Nichols, 2016). An important challenge for the future will be to better understand how abundance and structure of caveolae control the many functions currently ascribed to this intriguing class of membrane domains.

## Author contributions

Drafted the manuscript or revising it critically for important intellectual content: BH, AT, CC, AK. Approved the final version of the manuscript to be published: BH, AT, CC, AK. Agree to be accountable for all aspects of the work: BH, AT, CC, AK.

## Funding

Supported by NIH R01 HL111259 and R01 HL111259 01S1. The funding sources had no role in writing the report or the decision to submit the paper for publication.

### Conflict of interest statement

The authors declare that the research was conducted in the absence of any commercial or financial relationships that could be construed as a potential conflict of interest.
